# Impact of Antibiotic Stewardship on the Management of Pelvic Inflammatory Disease: A Multicenter Gynecological and Public Health Perspective in Pakistan

**DOI:** 10.7759/cureus.88739

**Published:** 2025-07-25

**Authors:** Hafiz Ali Shabbir Rajput, Muhammad Suffyan, Sehrish Jabeen, Maryam Noureen, Riffat Shaheen, Sana Noor, Sania Imran

**Affiliations:** 1 Department of Surgery, National Health Service, England, GBR; 2 Paediatrics and Neonatology, City Hospital, Multan, PAK; 3 Department of Anaesthesia, Combined Military Hospital Kharian, Kharian, PAK; 4 Department of Dermatology, Dr Rehmat Ullah Clinic, Depalpur, PAK; 5 Department of Obstetrics and Gynecology, Hayatabad Medical Complex Peshawar, Peshawar, PAK; 6 Department of Community Medicine, Avicenna Medical and Dental College and Hospital, Lahore, PAK; 7 Department of Obstetrics and Gynecology, Shalamar Hospital, Lahore, PAK

**Keywords:** antimicrobial stewardship, bacterial, drug resistance, gynecology, patient compliance, pelvic inflammatory disease (pid), recurrence, therapeutic use, treatment outcome

## Abstract

Background: Pelvic inflammatory disease (PID) is a significant gynecological and public health concern, frequently exacerbated by inappropriate antibiotic use and rising antimicrobial resistance.

Objective: This study aimed to evaluate the impact of an Antibiotic Stewardship Program (ASP) on individual clinical outcomes (recovery, recurrence, and treatment adherence) and broader public health indicators (antibiotic resistance trends, hospital admissions, and referrals) in the management of PID across multiple tertiary healthcare centers.

Materials and methods: This descriptive observational study was conducted at the Departments of Gynecology of the Pakistan Institute of Medical Sciences (PIMS), Islamabad; Shalamar Hospital, Lahore; City Hospital, Multan; Lady Reading Hospital (LRH), Peshawar; and Hayatabad Medical Complex (HMC), Peshawar, from August 2022 to July 2024. A total of 390 women aged 15-45 diagnosed with PID were enrolled - 190 prior to and 200 following the implementation of an ASP. Statistical analysis was performed using IBM SPSS Statistics for Windows version 26, employing descriptive statistics, Chi-square tests for categorical variables, and independent t-tests for continuous variables. A p-value of <0.05 was considered statistically significant.

Results: Following the intervention, treatment adherence significantly improved from 108 patients (56.84%) to 169 patients (84.50%) (p < 0.001). The number of patients experiencing symptom recurrence declined from 23 patients (12.11%) to nine patients (4.50%) (p = 0.014), while complete clinical recovery increased from 119 patients (62.63%) to 158 patients (79.00%) (p = 0.002). In addition, hospital admissions due to PID decreased from 73 patients (38.42%) to 49 patients (24.50%) (p = 0.006), and the average treatment duration was reduced from 11.6 days to 9.2 days (p < 0.001). Referrals related to antibiotic resistance also fell from 26 patients (13.68%) to 11 patients (5.50%) (p = 0.009). Although complications declined, the change was not statistically significant (p = 0.318).

Conclusion: Implementation of antibiotic stewardship across multiple tertiary care centers significantly improved clinical outcomes, enhanced treatment adherence, and contributed to a reduction in recurrence and resistance-associated referrals in PID management, supporting the value of coordinated antimicrobial policies from both gynecological and public health perspectives.

## Introduction

A major reproductive health problem, pelvic inflammatory disease (PID) affects millions of women worldwide and presents serious gynecological and public health issues [1,2]. PID, which is characterized by infection and inflammation of the upper female vaginal tract, is often caused by the ascent of sexually transmitted infections such as *Neisseria gonorrhoeae *and* Chlamydia trachomatis* [3]. Serious side effects such as infertility, ectopic pregnancy, and persistent pelvic discomfort may arise from PID if it is not treated or is not well controlled [4]. The burden is most severe in low- and middle-income nations, where the disease's long-term effects are made worse by a lack of access to prompt diagnosis and suitable treatment [5].

Empirical antibiotic treatment remains the cornerstone of PID management. However, its efficacy has increasingly been undermined by the rise of antimicrobial resistance (AMR), poor adherence to treatment guidelines, and the overprescription of broad-spectrum agents [6,7]. In response to these challenges, the integration of Antibiotic Stewardship Programs (ASPs) into clinical care has gained prominence, especially in settings where antibiotic misuse is widespread. ASPs are coordinated interventions designed to improve and measure the appropriate use of antimicrobials by promoting the selection of the optimal drug regimen, including dosing, duration, and route of administration, based on evidence and local susceptibility patterns [8]. In gynecological care, ASPs focus on standardizing PID treatment through evidence-based prescribing, resistance surveillance, clinician education, and multidisciplinary oversight to ensure effective and rational antibiotic use [9].

From the standpoint of public health, PID treatment is related to more general concerns about antimicrobial policy, reproductive health inequities, and STI monitoring [1,4]. In addition to contributing to the development of resistant organisms, the overuse of antibiotics in both inpatient and outpatient settings raises healthcare expenses and jeopardizes the effectiveness of subsequent treatments [10,11]. PID is thus a crucial paradigm for evaluating the effects of stewardship tactics in systemic and clinical settings.

There is a notable lack of region-specific data from low- and middle-income countries, including Pakistan, where the burden of PID is compounded by limited resources, diagnostic delays, and inconsistent treatment practices. In addition, few studies have systematically examined the dual impact of ASPs on both clinical recovery and broader public health indicators in gynecological settings. Even though ASPs are becoming more and more popular, little is known about how exactly they affect PID outcomes, especially in situations when healthcare systems are already struggling. Understanding how stewardship initiatives influence PID patient outcomes, microbial resistance patterns, and treatment practices is crucial for creating long-lasting and effective reproductive health policy. This study aimed to evaluate the impact of an ASP on both individual clinical outcomes (recovery, recurrence, and treatment adherence) and public health indicators (antibiotic resistance trends, hospital admissions, and referrals) in the management of PID.

## Materials and methods

Study design and setting

This descriptive observational study was conducted at the Departments of Gynecology of the Pakistan Institute of Medical Sciences (PIMS), Islamabad; Shalamar Hospital, Lahore; City Hospital, Multan; Lady Reading Hospital (LRH), Peshawar; and Hayatabad Medical Complex (HMC), Peshawar, from August 2022 to July 2024. A multi-center observational design was selected, as it reflects clinical practice and is recommended for evaluating antimicrobial stewardship interventions in diverse settings where randomized trials are impractical or unethical [12-15]. These hospitals were purposefully selected to represent diverse urban populations, healthcare practices, and microbiological capacities across four provinces, thereby increasing the external validity and generalizability of our findings. The study evaluated the impact of an ASP on both individual clinical outcomes (recovery, recurrence, adherence, and treatment duration) and public health indicators (antibiotic resistance trends, hospital admissions, and referrals) in the management of PID.

Inclusion and exclusion criteria

The study included women aged 15-45 years diagnosed with PID based on a combination of clinical, laboratory, and imaging findings, consistent with the Centers for Disease Control (CDC) diagnostic criteria for PID [16]. Eligible participants were identified from both inpatient and outpatient settings after providing written informed consent. A clinical diagnosis of PID requires at least one of the following minimum criteria in the presence of lower abdominal or pelvic pain: cervical motion tenderness, uterine tenderness, or adnexal tenderness. Supportive criteria included fever >38°C, leukocytosis, elevated inflammatory markers (CRP or ESR), mucopurulent cervical discharge, or transvaginal ultrasound findings suggestive of thickened, fluid-filled fallopian tubes, tubo-ovarian complex, or free pelvic fluid. For microbiological confirmation, endocervical and high vaginal swabs were routinely collected. The most commonly identified pathogens in our cohort were *Neisseria gonorrhoeae*, *Chlamydia trachomatis*, *Mycoplasma genitalium*, *Escherichia coli*, *Gardnerella vaginalis*, *Bacteroides fragilis*, *Peptostreptococcus* spp., *Ureaplasma urealyticum*, and group B *Streptococcus*, reflecting the known polymicrobial etiology of PID in our regional context.

Patients were excluded if they had incomplete PID treatment from other hospitals, were pregnant, had known immunocompromising conditions such as HIV infection, or had pelvic pain attributed to non-infectious or non-gynecological causes such as endometriosis, ovarian cyst rupture, or appendicitis.

Sample size

We enrolled a total of 390 consecutive patients with PID, including 190 in the pre-intervention group and 200 in the post-intervention group. The sample size was calculated based on detecting a clinically meaningful improvement in adherence rates of at least 20 percentage points (from ~57% to ~77%) between groups, with a two-sided significance level of 5% and power of 80%.

We applied the standard formula for comparing two independent proportions, widely used in clinical research design [17]:

$n = \frac{(Z_{1-\alpha/2} + Z_{1-\beta})^{2} . [p_{1}(1&minus;p_{1})+p_{2}(1&minus;p_{2})]}{(p_{1} - p_{2})}$,

where *p_1_* and *p_2_* are the expected proportions in the two groups, *α* is the type I error probability, and *β* is the type II error probability. Using *p_1 _*​= 0.57, *p_2 _*= 0.77, *α *= 0.05, and *β *= 0.20, the calculated minimum required sample size per group was approximately 83 patients. Our final sample of nearly 200 patients per group exceeded this requirement more than twofold, ensuring robust statistical power to detect significant differences. This aligns with and exceeds sample sizes reported in similar multi-center observational ASP studies, including a systematic review and meta-analysis by Schuts et al. [18]. Recruiting consecutive patients over two years further minimized selection bias and improved representativeness.

Data collection

Data were collected using a standardized proforma designed to capture patient demographics, clinical presentation, diagnostic findings, antibiotic regimens, compliance with prescribed treatment protocols, therapeutic response, and follow-up outcomes. To minimize interobserver variability, data collectors at each site were trained on the use of the standardized proforma, and periodic cross-site monitoring was performed by the central coordinating team. Patients were monitored for up to three months after initiating therapy to assess clinical recovery, recurrence of symptoms, and any complications. The ASP was formally implemented at the beginning of the second study year (August 2023), enabling a clear comparison between pre-intervention and post-intervention groups. Key ASP interventions included dissemination of updated PID management guidelines [19], structured educational sessions for prescribers, regular audits of antibiotic prescriptions with feedback, and integration of AMR surveillance into clinical decision-making. The implementation and impact of stewardship protocols, such as guideline-concordant prescribing, AMR monitoring, and physician education, were evaluated through patient records and institutional audit data. Adherence to the ASP protocol (including audit-feedback cycles and prescriber training) was tracked through site-specific logs and monthly review meetings to ensure consistent implementation across all centers.

In addition to clinical, laboratory, and microbiological data, a structured patient-reported outcomes questionnaire was administered at the three-month follow-up visit. The questionnaire (see Appendix A) was adapted and developed specifically for this study, based on the validated instrument by Park et al. [20], which assessed patient awareness, knowledge, and perceptions regarding appropriate antibiotic use and the need for outpatient antimicrobial stewardship programs. The original questionnaire items were reviewed by the author team and discussed in detail to identify domains relevant to the management of PID in our multi-center Pakistani setting. Modifications were made through consensus to include additional items on self-reported adherence to PID treatment, persistence or recurrence of symptoms, satisfaction with care, perceived barriers to adherence, and patient knowledge and attitudes regarding antibiotic use, while ensuring cultural and contextual appropriateness.

Sexual activity history was self-reported and categorized as “recent” if intercourse occurred within the past six months. Participants reporting no recent activity included a majority of married women abstaining due to personal, cultural, or religious reasons, and a minority of unmarried women who had never been sexually active. In Pakistani society, strongly influenced by Islamic values, premarital sexual activity is generally discouraged, which may explain higher rates of sexual inactivity reported by some participants. While PID is predominantly linked to sexually transmitted pathogens, non-sexually active women were not excluded, acknowledging that PID can also arise from non-sexual routes such as ascending infections due to endogenous flora, gynecological procedures, or poor menstrual hygiene. This cultural and biological context was considered both in data interpretation and in adapting the patient questionnaire to ensure its relevance, sensitivity, and acceptability within the study population.

Microbiological analysis

Microbiological testing was performed on specimens collected aseptically from all enrolled patients at the time of diagnosis. These included endocervical swabs, high vaginal swabs, and, where clinically indicated, endometrial aspirates or drained material from tubo-ovarian abscesses. The diagnostic approach prioritized cost-effective and widely accessible techniques, making it feasible across all participating hospitals in Pakistan. Gram staining was used as an initial screening tool to detect polymorphonuclear leukocytes, identify gram-negative intracellular diplococci suggestive of Neisseria gonorrhoeae, and assess the presence of mixed flora indicative of anaerobic infections.

Pathogen isolation was performed using standard culture methods. Thayer-Martin selective agar was used for isolating *N. gonorrhoeae*, while blood and MacConkey agar were utilized to identify facultative organisms such as *E. coli* and group B* Streptococcus*. For anaerobic organisms, anaerobic culture media supported the growth of *Bacteroides fragilis* and *Peptostreptococcus* spp. Although nucleic acid amplification tests (NAATs) for *Chlamydia trachomatis* and *Mycoplasma genitalium *were not universally available, they were used selectively in better-equipped laboratories using PCR-based methods or enzyme immunoassays. The diagnostic strategy reflected the real-world limitations of public hospital infrastructure, relying primarily on culture and microscopy to ensure broad pathogen coverage at low cost.

The most frequently identified pathogens in this study included *N. gonorrhoeae*, *C. trachomatis*, *M. genitalium*, *E. coli*, *Gardnerella vaginalis*, *B. fragilis*, *Peptostreptococcus* spp., *Ureaplasma urealyticum*, and group B *Streptococcus*. Antibiotic susceptibility testing was carried out using the Kirby-Bauer disk diffusion method, interpreted according to Clinical and Laboratory Standards Institute (CLSI) guidelines. Automated systems, such as VITEK 2, were employed to complement manual testing, but were not used routinely due to cost considerations. This microbiological approach allowed for consistent identification of pathogens and resistance profiles across centers, while remaining aligned with the diagnostic capabilities of secondary and tertiary hospitals in Pakistan.

Statistical analysis

All data were entered and analyzed using IBM SPSS Statistics for Windows, version 26.0 (released 2018, IBM Corp., Armonk, NY). Descriptive statistics, including mean ± standard deviation for continuous variables and frequencies with percentages for categorical variables, were used to summarize baseline characteristics. For pre- and post-intervention comparisons, Chi-square tests were used for categorical variables (e.g., adherence rates, recovery rates, and hospital admissions), and independent samples t-tests were applied for continuous variables (e.g., treatment duration). To adjust for potential confounding factors and assess the independent effect of the ASP intervention, multivariable logistic regression analyses were performed, including variables such as age, marital status, comorbidities, baseline severity of symptoms, and microbiological findings. A p-value of less than 0.05 was considered statistically significant.

Ethical approval

The study protocol was reviewed and approved by the Hospital Research and Ethical Committee, MTI - Hayatabad Medical Complex, Peshawar, Pakistan (No. 424; date: August 2, 2022). Written informed consent was obtained from all participants prior to enrollment.

## Results

The baseline clinical and demographic characteristics of 390 PID patients are presented in Table 1. Most patients were between the ages of 25 and 34 (n = 171, 43.85%), followed by those between the ages of 35 and 45 (n = 121, 31.03%) and 15 and 24 (n = 98, 25.13%). Lower abdomen discomfort (n = 342, 87.69%), abnormal vaginal discharge (n = 289, 74.10%), fever (n = 204, 52.31%), dyspareunia (n = 126, 32.31%), and irregular bleeding (n = 78, 20.00%) were the most prevalent clinical complaints, and the majority were married (n = 341, 87.44%).

**Table 1:** Baseline demographic and clinical characteristics of the patients with pelvic inflammatory disease (PID) (N = 390).

Variable	Subcategory	Number of patients (n, %)
Age group (years)	15–24	98 (25.13%)
	25–34	171 (43.85%)
	35–45	121 (31.03%)
Marital status	Married	341 (87.44%)
	Unmarried	49 (12.56%)
Clinical presentation	Lower abdominal pain	342 (87.69%)
	Abnormal vaginal discharge	289 (74.10%)
	Fever	204 (52.31%)
	Dyspareunia	126 (32.31%)
	Irregular bleeding	78 (20.00%)

**Figure 1:**
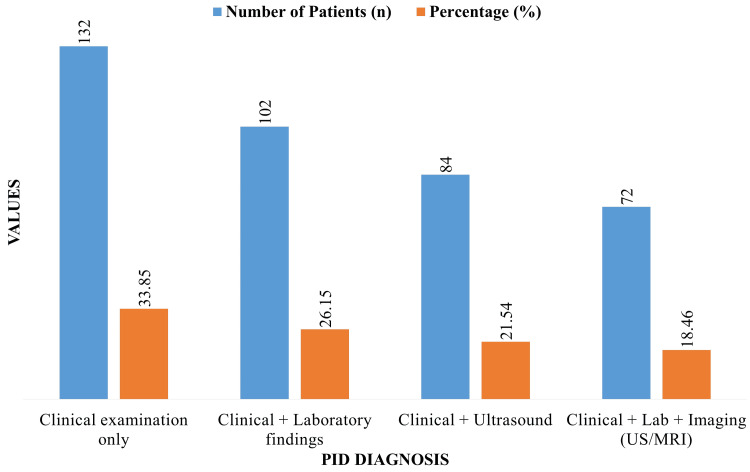
Diagnostic modalities utilized for pelvic inflammatory disease (PID) diagnosis.

As shown in Figure 1, clinical examination alone was performed on 132 patients (33.85%), combined clinical and lab results were performed on 102 patients (26.15%), clinical plus ultrasound was performed on 84 patients (21.54%), and comprehensive assessment with clinical, lab, and imaging was performed on 72 patients (18.46%).

The pre-intervention group (n = 190) and post-intervention group (n = 200) were largely comparable (Table 2). The mean age was 28.9 ± 6.4 years for the pre-intervention group and 29.2 ± 6.1 years for the post-intervention group (p = 0.62). The median parity was 2 (IQR: 1-3) in both groups (p = 0.88). A similar proportion of patients were managed as inpatients in the pre- (51.6%, n = 98) and post-intervention groups (53.0%, n = 106; p = 0.78). Prior history of PID - referring specifically to documented episodes before the current presentation - was reported by 12.6% (n = 24) of the pre-intervention group and 14.5% (n = 29) of the post-intervention group (p = 0.65). The mean symptom duration before presentation was 6.2 ± 3.1 days in the pre-intervention group and 6.0 ± 3.3 days in the post-intervention group (p = 0.71). Screening for sexually transmitted infections (STIs) was conducted in 38.9% (n = 74) and 40.0% (n = 80) of patients in the pre- and post-intervention groups, respectively. Sexual activity in the month prior to presentation was reported by 71.1% (n = 135) and 69.5% (n = 139) of patients in the two groups (p = 0.80). No statistically significant differences were found, confirming baseline comparability for outcome analyses.

**Table 2:** Comparison of baseline characteristics between pre- and post-intervention groups. SD = standard deviation; IQR = interquartile range; t = independent samples t-test statistic; χ² = Chi-square test statistic; df = degrees of freedom; p-value <0.05 was set as significant.

Variable	Pre-intervention (n = 190)	Post-intervention (n = 200)	Statistical tests (df)	p-value
Age (mean ± SD)	28.9 ± 6.4	29.2 ± 6.1	t(388)=−0.50	0.62
Parity (median, IQR)	2 (1–3)	2 (1–3)	χ²(1)=0.02	0.88
Inpatient cases (%)	98 (51.6%)	106 (53.0%)	χ²(1)=0.08	0.78
Prior PID episode (%)	24 (12.6%)	29 (14.5%)	χ²(1)=0.20	0.65
Duration of symptoms (mean ± SD)	6.2 ± 3.1	6.0 ± 3.3	t(388)=0.38	0.71
Sexual activity reported (%)	135 (71.1%)	139 (69.5%)	χ²(1)=0.06	0.8
STI screening performed (%)	74 (38.9%)	80 (40.0%)	χ²(1)=0.04	0.83

Following implementation of the ASP, there was a statistically significant improvement in adherence to recommended antibiotic regimens (Table 3). The number of patients receiving ceftriaxone plus doxycycline increased from 87/190 (45.79%) in the pre-intervention group to 106/200 (53.00%) in the post-intervention group. Similarly, cefoxitin plus probenecid use increased from 43/190 (22.63%) to 56/200 (28.00%), leading to a combined increase from 130/190 (68.42%) to 162/200 (81.00%) in guideline-concordant prescribing (p < 0.05). These two combinations were preferred in the ASP due to their effectiveness against common PID pathogens and local resistance data. Concurrently, the use of non-preferred regimens, such as clindamycin plus gentamicin or metronidazole plus doxycycline, decreased, indicating a shift toward more appropriate prescribing. However, 38/200 (19.00%) of cases post-intervention still involved non-concordant regimens, largely due to documented allergies, intolerance, or prior treatment failure.

**Table 3:** Comparison of antibiotic regimens used before and after Antibiotic Stewardship Program (ASP) implementation.

Regimen type	Pre-intervention (n = 190)	Post-intervention (n = 200)
Ceftriaxone + doxycycline	87 (45.79%)	106 (53.00%)
Cefoxitin + probenecid	43 (22.63%)	56 (28.00%)
Clindamycin + gentamicin	26 (13.68%)	18 (9.00%)
Metronidazole + doxycycline	21 (11.05%)	15 (7.50%)
Other combinations	13 (6.84%)	5 (2.50%)

**Figure 2:**
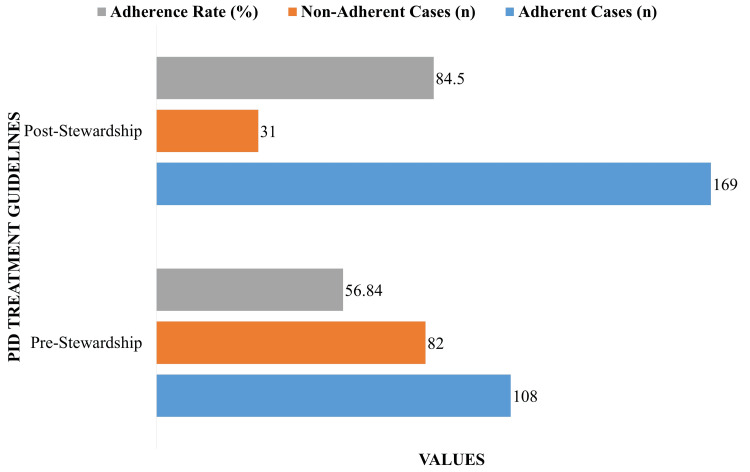
Adherence to pelvic inflammatory disease (PID) treatment guidelines.

Figure 2 demonstrates compliance with PID treatment recommendations before and after the implementation of the ASP. Prior to ASP, 108 patients (56.84%) received treatment aligned with institutional guidelines, while 82 cases showed non-adherence. These guidelines, although informally followed, were not standardized or provided in written form to patients. After ASP implementation, compliance rose markedly, with 169 patients (84.50%) receiving guideline-concordant treatment, and instances of non-adherence fell significantly to 31. This improvement reflects the structured introduction of written treatment protocols, enhanced prescriber training, and clearer patient communication, all of which contributed to increased adherence and consistency in care delivery.

The resistance rates of isolated pathogens are listed in Table 4. The bacteria that showed the highest resistance were *N. gonorrhoeae* (32 cases, 8.21%), *E. coli* (29 cases, 7.44%), *Chlamydia trachomatis* (21 cases, 5.38%), and *Mycoplasma genitalium* (17 cases, 4.36%), raising new worries about antibiotic resistance.

**Table 4:** Antimicrobial resistance trends in isolated pathogens.

Pathogen	Total isolates (n)	Resistant to ≥1 drug (n)	Resistance rate (%)
Neisseria gonorrhoeae	84	32	38.10%
Escherichia coli	74	29	39.19%
Chlamydia trachomatis	61	21	34.43%
Mycoplasma genitalium	59	17	28.81%
Gardnerella vaginalis	24	5	20.83%
Bacteroides fragilis	19	6	31.58%
*Peptostreptococcus *spp.	15	4	26.67%
Ureaplasma urealyticum	11	3	27.27%
Group B *Streptococcus*	10	2	20.00%

Following the implementation of the ASP, significant improvements were observed in both clinical and system outcomes (Table 5). Adherence to treatment guidelines increased from 56.84% to 84.50% (p < 0.001; 95% CI: 18.9% to 37.5%), while complete clinical recovery improved from 62.63% to 79.00% (p = 0.002; 95% CI: 6.4% to 26.1%). Hospital admissions for PID declined from 73 (38.42%) to 49 (24.50%) (−32.88%), the average treatment duration decreased from 11.6 to 9.2 days (−20.69%), and the number of referrals owing to antibiotic resistance decreased from 26 (13.68%) to 11 (5.50%) (−57.69%). Although the complication rate decreased from 3.68% to 2.00%, this change was not statistically significant (p = 0.318; 95% CI: −4.9% to 1.5%); however, the trend may still hold clinical importance given the overall improvement in outcomes.

**Table 5:** Comparative analysis of clinical and system outcomes before and after antibiotic stewardship implementation. CI = confidence interval; df = degrees of freedom; PID = pelvic inflammatory disease; t = independent samples t-test statistic; χ² = Chi-square test statistic.

Outcome parameter	Pre-stewardship (n = 190)	Post-stewardship (n = 200)	Statistical test	p-value	95% CI (post − pre)
Complete clinical recovery (%)	119 (62.63%)	158 (79.00%)	χ² = 9.84	0.002	6.4% to 26.1%
Partial improvement (%)	41 (21.58%)	29 (14.50%)	χ² = 6.67	0.009	2.5% to 15.1%
Recurrence of symptoms (%)	23 (12.11%)	9 (4.50%)	χ² = 6.01	0.014	−13.7% to −1.6%
Development of complications (%)	7 (3.68%)	4 (2.00%)	χ² = 1.00	0.318	−4.9% to 1.5%
Adherence to treatment guidelines (%)	108 (56.84%)	169 (84.50%)	χ² = 33.67	<0.001	18.9% to 37.5%
Average treatment duration (days)	11.6 ± 2.4	9.2 ± 2.1	t= 11.34	<0.001	−2.97 to −1.43
Hospital admissions for PID (%)	73 (38.42%)	49 (24.50%)	χ² = 7.52	0.006	−23.7% to −4.2%
Referred due to resistance (%)	26 (13.68%)	11 (5.50%)	χ² = 6.79	0.009	−14.2% to −2.2%

## Discussion

PID is still a major clinical and public health issue, especially in places with low resources, where improper antibiotic usage and delayed diagnosis lead to poor reproductive outcomes and an increase in AMR. Real-world adherence to set norms is often deficient. ASPs provide a methodical way to maximize the usage of antibiotics and enhance results. This research highlights the efficacy of an ASP in controlling PID by assessing its effects in a high-volume gynecological practice.

The results of this research highlight how ASPs significantly improve PID clinical care and public health outcomes. Adherence to suggested treatment parameters increased significantly from 56.84% before the intervention to 84.50% after the introduction of stewardship measures. This is in line with other research that shows that following established PID treatment guidelines is linked to better clinical results and fewer problems [21,22]. Complete clinical recovery improved significantly, rising from 62.63% before the intervention to 79.00% after it. This is consistent with other research that showed a high percentage of recovery when rigorously adhering to guidelines-directed treatment [23].

The impact of the ASP is further demonstrated by the shift in antibiotic prescribing trends. Ceftriaxone + doxycycline remained the most frequently recommended regimen in both phases, increasing from 45.79% to 53.00%, reflecting improved adherence to evidence-based protocols. Meanwhile, the use of alternative regimens such as metronidazole + doxycycline and clindamycin + gentamicin declined significantly. Although these combinations were not entirely eliminated, their reduction highlights enhanced diagnostic precision and antibiotic selection post-ASP implementation. The ASP did not categorically prohibit their use, as they may still be appropriate in specific clinical contexts; for example, clindamycin + gentamicin remains an acceptable regimen for severe PID in hospitalized patients allergic to β-lactams, and metronidazole + doxycycline may be used in resource-limited settings or mild cases without gonorrheal co-infection. Therefore, the stewardship strategy emphasized rational use based on clinical presentation, resource availability, and pathogen prevalence rather than strict exclusion. This tailored approach aligns with real-world practice and mirrors findings from prior ASP studies showing that context-specific prescribing, rather than rigid protocols, leads to sustained reductions in inappropriate antibiotic use [24].

Significant reductions in hospital admissions for PID (38.42% to 24.50%, p = 0.006) and AMR-related referrals (13.68% to 5.50%, p = 0.009) further demonstrate the effectiveness of stewardship initiatives in reducing resistance-related consequences and healthcare burden. Prior research studies showed similar results, showing a substantial decrease in hospitalizations associated with PID [4,25].

Although concerning, the resistance rates observed in this study, i.e., 8.21% for *N. gonorrhoeae* and 7.44% for *E. coli*, are consistent with global trends that underscore the growing challenge of antimicrobial resistance in urogenital infections [26,27]. These findings reinforce the need for regional antimicrobial stewardship adaptations and continuous microbiological surveillance. Notably, the average duration of antibiotic therapy significantly decreased from 11.6 to 9.2 days (p < 0.001), reflecting a shift toward more streamlined, guideline-concordant treatment. This reduction was achieved through targeted interventions of the ASP, including regular audit and feedback sessions, prescriber education on PID-specific regimens, development of simplified treatment algorithms, and reinforcement of evidence-based duration limits. Importantly, the shorter treatment duration did not compromise patient outcomes, as evidenced by increased recovery rates and decreased recurrence and referral rates. These results demonstrate that structured ASPs can optimize antibiotic use, enhance patient outcomes, and yield broader public health benefits by minimizing resistance development, reducing unnecessary drug exposure, and improving healthcare resource utilization.

Strengths and limitations

This multi-center, descriptive observational study conducted over two years at five major tertiary care hospitals in Pakistan represents a significant strength, as it reflects real-world clinical practices across diverse settings while enabling structured and comprehensive data collection. The inclusion of both inpatient and outpatient cases and the recruitment of consecutive patients helped reduce selection bias and enhance the representativeness of the sample. The relatively large sample size (n = 390) exceeded the minimum required size based on power calculations, ensuring robust statistical power to detect clinically meaningful differences in outcomes. Furthermore, baseline comparability between the pre- and post-intervention groups was formally assessed and confirmed, strengthening the internal validity of comparisons. A major methodological strength of this study was its comprehensive evaluation of the ASP by integrating multiple outcome measures at both the individual level (clinical recovery, adherence, recurrence, complications) and the public health level (antimicrobial resistance trends, hospital admissions, and AMR-related referrals). Standardized data collection tools, laboratory testing aligned with CLSI guidelines, and consistent follow-up at three months further support the reliability and validity of findings. Steps to minimize bias included consecutive sampling, uniform diagnostic and follow-up protocols across centers (see Appendix B), and statistical testing to confirm baseline group equivalence.

However, the observational nature of the study inherently limits causal inference, as potential confounding variables cannot be fully controlled, and randomization was not feasible due to ethical and practical constraints. Although the use of consecutive sampling reduced some bias, it is still a non-probability sampling method, which may affect generalizability. The setting, limited to tertiary care hospitals in Pakistan, may also restrict external validity in other healthcare environments, particularly primary care or rural contexts. Additionally, while microbiological cultures and susceptibility testing were performed on all enrolled patients, the proportion of culture-positive cases was relatively low, limiting pathogen-specific outcome analyses. The three-month follow-up period, though pragmatic, may have been insufficient to fully capture long-term outcomes such as chronic pelvic pain, infertility, or recurrent PID beyond this window. Finally, while a formal sample size calculation was conducted and exceeded, the absence of multivariable adjustment for potential confounders is another limitation to consider. Future research incorporating longer follow-up, adjustment for confounding factors, and randomized or controlled designs, where feasible, would further strengthen the evidence on ASP effectiveness in PID management.

## Conclusions

This research shows that the clinical treatment and public health outcomes of PID are greatly improved by the use of antibiotic stewardship strategies. Following the intervention, there was a decrease in recurrence, treatment duration, hospitalizations, and referrals due to antibiotic resistance, as well as increased adherence to treatment recommendations (84.50% vs. 56.84%) and improved full recovery. These results highlight the importance of ASPs in enhancing patient outcomes, maximizing the use of antibiotics, and preventing the emergence of resistance in reproductive health settings. The findings back up the inclusion of stewardship procedures as a crucial aspect of PID treatment plans, especially in hospital institutions with limited resources.

## Appendices

Appendix A 

**Table 6:** Patient questionnaire for pelvic inflammatory disease (PID)–Antibiotic Stewardship Program (ASP) Study. Source: Park et al. [20]

Domain	Question / variable	Response options / scale
Demographics	Age	___ years
	Marital status	☐ Married ☐ Unmarried
	Education level	☐ None ☐ Primary ☐ Secondary ☐ Higher
	Occupation	☐ Housewife ☐ Employed ☐ Student ☐ Other: _____
Knowledge about antibiotics	Do you believe antibiotics are always necessary for vaginal/abdominal infections?	☐ Yes ☐ No ☐ Don’t know
	Have you ever taken leftover antibiotics from a previous illness?	☐ Yes ☐ No
	Do you think it’s okay to stop antibiotics when symptoms improve?	☐ Yes ☐ No ☐ Not sure
	Do you know what “antibiotic resistance” means?	☐ Yes ☐ No
Treatment adherence (self-reported)	Did you complete the full course of antibiotics as prescribed?	☐ Yes ☐ No
	If no, what was the main reason?	☐ Side effects ☐ Felt better ☐ Forgot ☐ Couldn’t afford ☐ Other: ___
Current symptoms (at follow-up)	Lower abdominal pain	☐ None ☐ Mild ☐ Moderate ☐ Severe
	Abnormal vaginal discharge	☐ Yes ☐ No
	Fever in the past 7 days	☐ Yes ☐ No
	Pain during intercourse (dyspareunia)	☐ Yes ☐ No
	Irregular bleeding	☐ Yes ☐ No
Satisfaction and experience	Were you satisfied with the doctor’s explanation about your illness and treatment?	☐ Very satisfied ☐ Satisfied ☐ Neutral ☐ Dissatisfied ☐ Very dissatisfied
	How easy was it to follow the treatment instructions?	☐ Very easy ☐ Easy ☐ Neutral ☐ Difficult ☐ Very difficult
	Would you recommend this hospital to others for such care?	☐ Yes ☐ No ☐ Not sure
	Were the staff respectful and supportive during your treatment?	☐ Yes ☐ No
Health-seeking behavior	Did you visit another hospital/clinic for this illness during follow-up?	☐ Yes ☐ No
	If yes, why?	☐ Symptoms worsened ☐ Not satisfied here ☐ Referred ☐ Other: ___
Open feedback (optional)	What suggestions do you have to improve care for women with this illness?	___________________________

Appendix B 

**Table 7:** Bias minimization strategies employed in the study.

Strategy	Implementation details	Rationale and impact on study validity
Consecutive sampling	All eligible patients diagnosed with PID during pre- and post-ASP periods were enrolled consecutively.	Avoided selection bias; ensured representative sampling across both cohorts (n = 190 vs. 200).
Uniform diagnostic criteria	Standardized diagnostic approach per CDC/WHO criteria using clinical signs, laboratory markers, and imaging, where applicable.	Enhanced diagnostic reliability; minimized variability between pre- and post-ASP diagnoses.
Standardized follow-up protocol	Fixed follow-up at day 7–10 with clearly defined criteria for recovery, recurrence, or complications.	Ensured consistent measurement of outcomes like complete recovery (62.6% → 79.0%) and recurrence (12.1% → 4.5%).
Unified treatment guidelines	Post-ASP, clinicians followed a common antimicrobial policy with updates and training based on resistance patterns and international recommendations.	Boosted treatment uniformity and led to increased adherence (56.8% → 84.5%, p<0.001).
Blinded outcome assessment	Outcome assessors were not involved in the prescription or management of patients.	Reduced observer bias in assessing subjective outcomes.
Audit and feedback loop	ASP team conducted monthly prescription audits and shared performance feedback with care teams.	Promoted guideline adherence and contributed to systemic improvements (e.g., hospital admissions: 38.4% → 24.5%, p=0.006).
Same institutional setting	Study conducted in the same hospital and departments across both periods.	Controlled for institutional variation and confounders related to staffing or protocols.
Antibiotic resistance monitoring	Pre- and post-ASP pathogen cultures and susceptibility testing were routinely performed.	Supported rational drug choices and demonstrated reduced resistance-driven referrals (13.7% → 5.5%, p=0.009).
Appropriate statistical methods	Chi-square, t-tests, and confidence intervals used to compare groups; significance set at p < 0.05.	Confirmed statistical validity and minimized risk of random error.
Data validation and completeness	Electronic medical records and pharmacy logs were cross-verified for completeness and consistency.	Improved data quality and reliability of outcome reporting.

## References

[1] Hillier SL, Bernstein KT, Aral S (2021). A review of the challenges and complexities in the diagnosis, etiology, epidemiology, and pathogenesis of pelvic inflammatory disease. J Infect Dis.

[2] He D, Wang T, Ren W (2023). Global burden of pelvic inflammatory disease and ectopic pregnancy from 1990 to 2019. BMC Public Health.

[3] Mohamed AA (2024). Pelvic inflammatory disease: clinical feature, risk factors, treatment, and prevention. Indian J Comm Health.

[4] Yusuf H, Trent M (2023). Management of pelvic inflammatory disease in clinical practice. Ther Clin Risk Manag.

[5] Brunham RC, Gottlieb SL, Paavonen J (2015). Pelvic inflammatory disease. N Engl J Med.

[6] Sultana A, Mehdi S, Rahman K, Fazmiya MJ, Heyat MB, Akhtar F, Baig AA (2022). Recent advancements of pelvic inflammatory disease: a review on evidence-based medicine. Computational Intelligence in Healthcare Applications.

[7] Frock-Welnak DN, Tam J (2022). Identification and treatment of acute pelvic inflammatory disease and associated sequelae. Obstet Gynecol Clin North Am.

[8] Rajput A (2023). Examining the implementation and effectiveness of antibiotic stewardship programs in healthcare settings to prevent antibiotic resistance and promote prudent antibiotic use. Knowledgeable Res.

[9] Majumder MA, Rahman S, Cohall D, Bharatha A, Singh K, Haque M, Gittens-St Hilaire M (2020). Antimicrobial stewardship: fighting antimicrobial resistance and protecting global public health. Infect Drug Resist.

[10] Oyedum UM, Kuta FA, Saidu AN, Babayi H (2023). Survey of multidrug resistant Salmonella enterica serovar typhi from patients with pelvic inflammatory disease attending some hospitals in Niger State, Nigeria. UMYU J Microbiol Res.

[11] Ness RB, Soper DE, Holley RL (2002). Effectiveness of inpatient and outpatient treatment strategies for women with pelvic inflammatory disease: results from the Pelvic Inflammatory Disease Evaluation and Clinical Health (PEACH) Randomized Trial. Am J Obstet Gynecol.

[12] Desai V, Kumar S, Patel B (2025). Navigating antimicrobials and combating antimicrobial resistance: challenges, impacts, and strategies for global action. Cureus.

[13] Schweitzer VA, van Heijl I, van Werkhoven CH (2019). The quality of studies evaluating antimicrobial stewardship interventions: a systematic review. Clin Microbiol Infect.

[14] Bankar NJ, Ugemuge S, Ambad RS, Hawale DV, Timilsina DR (2022). Implementation of antimicrobial stewardship in the healthcare setting. Cureus.

[15] (2025). CDC core elements of hospital antibiotic stewardship programs. https://www.cdc.gov/antibiotic-use/hcp/core-elements/hospital.html.

[16] (2025). Pelvic inflammatory disease (PID). https://www.cdc.gov/std/treatment-guidelines/pid.htm.

[17] Lwanga SK, Lemeshow S, World Health Organization (1991). Sample size determination in health studies: a practical manual. a practical manual / S. K. Lwanga.

[18] Schuts E, Hulscher ME, Mouton J (2016). Current evidence on hospital antimicrobial stewardship objectives: a systematic review and meta-analysis. Lancet Infect Dis.

[19] Workowski KA, Bachmann LH, Chan PA (2021). Sexually transmitted infections treatment guidelines, 2021. MMWR Recomm Rep.

[20] Park S, Geum MJ, Choi HJ, Kim CJ, Kwack WG, Chung EK, Rhie SJ (2021). Validation of a questionnaire for patient awareness and the need for a community-based Outpatient Antimicrobial Stewardship Program (O-ASP): a pilot study. Antibiotics (Basel).

[21] Das BB, Ronda J, Trent M (2016). Pelvic inflammatory disease: improving awareness, prevention, and treatment. Infect Drug Resist.

[22] Shih TY, Gaydos CA, Rothman RE, Hsieh YH (2011). Poor provider adherence to the Centers for Disease Control and Prevention treatment guidelines in US emergency department visits with a diagnosis of pelvic inflammatory disease. Sex Transm Dis.

[23] Haggerty CL, Ness RB (2008). Diagnosis and treatment of pelvic inflammatory disease. Womens Health (Lond).

[24] Malhotra M, Sharma JB, Batra S, Arora R, Sharma S (2023). Ciprofloxacin-tinidazole combination, fluconazole-azithromicin-secnidazole-kit and doxycycline-metronidazole combination therapy in syndromic management of pelvic inflammatory disease: a prospective randomized controlled trial. Indian J Med Sci.

[25] Sørbye IK, Jerve F, Staff AC (2005). Reduction in hospitalized women with pelvic inflammatory disease in Oslo over the past decade. Acta Obstet Gynecol Scand.

[26] Omeershffudin UN, Kumar S (2023). Emerging threat of antimicrobial resistance in Neisseria gonorrhoeae: pathogenesis, treatment challenges, and potential for vaccine development. Arch Microbiol.

[27] Pestrikova T, Yurasova E, Yurasov I (2019). A strategy of antimicrobial therapy of pelvic inflammatory diseases at antibiotic resistance of microbial pathogens. Gynecology.

